# Applying Machine Learning Algorithms for the Classification of Mink Infected with Aleutian Disease Using Different Data Sources

**DOI:** 10.3390/ani12182386

**Published:** 2022-09-13

**Authors:** Duy Ngoc Do, Guoyu Hu, Pourya Davoudi, Alimohammad Shirzadifar, Ghader Manafiazar, Younes Miar

**Affiliations:** Department of Animal Science and Aquaculture, Dalhousie University, Truro, NS B2N 5E3, Canada

**Keywords:** Aleutian disease, classification, machine learning, mink, random forest

## Abstract

**Simple Summary:**

Aleutian disease (AD) is a major infectious disease found in mink farms, and it causes financial losses to the mink industry. Controlling AD often requires a counterimmunoelectrophoresis (CIEP) method, which is relatively expensive for mink farmers. Therefore, predicting AD infected mink without using CIEP records will be important for controlling AD in mink farms. In the current study, we applied nine machine learning algorithms to classify AD-infected mink. We indicated that the random forest could be used to classify AD-infected mink (accuracy of 0.962) accurately. This result could be used for implementing machine learning in controlling AD in the mink farms.

**Abstract:**

American mink (*Neogale vison*) is one of the major sources of fur for the fur industries worldwide, whereas Aleutian disease (AD) is causing severe financial losses to the mink industry. A counterimmunoelectrophoresis (CIEP) method is commonly employed in a test-and-remove strategy and has been considered a gold standard for AD tests. Although machine learning is widely used in livestock species, little has been implemented in the mink industry. Therefore, predicting AD without using CIEP records will be important for controlling AD in mink farms. This research presented the assessments of the CIEP classification using machine learning algorithms. The Aleutian disease was tested on 1157 individuals using CIEP in an AD-positive mink farm (Nova Scotia, Canada). The comprehensive data collection of 33 different features was used for the classification of AD-infected mink. The specificity, sensitivity, accuracy, and F1 measure of nine machine learning algorithms were evaluated for the classification of AD-infected mink. The nine models were artificial neural networks, decision tree, extreme gradient boosting, gradient boosting method, K-nearest neighbors, linear discriminant analysis, support vector machines, naive bayes, and random forest. Among the 33 tested features, the Aleutian mink disease virus capsid protein-based enzyme-linked immunosorbent assay was found to be the most important feature for classifying AD-infected mink. Overall, random forest was the best-performing algorithm for the current dataset with a mean sensitivity of 0.938 ± 0.003, specificity of 0.986 ± 0.005, accuracy of 0.962 ± 0.002, and F1 value of 0.961 ± 0.088, and across tenfold of the cross-validation. Our work demonstrated that it is possible to use the random forest algorithm to classify AD-infected mink accurately. It is recommended that further model tests in other farms need to be performed and the genomic information needs to be used to optimize the model for implementing machine learning methods for AD detection.

## 1. Introduction

Mink is the major source of the fur industry worldwide [1], and Aleutian disease (AD), which is caused by the Aleutian mink disease virus (AMDV), brings tremendous financial losses to the mink industry [2]. The AD is associated with some important traits of farmed mink, including reproductive performance [3,4], body size [5], feed intake [6,7], and pelt quality [8]. There are several methods to test for AD, including the AMDV antigen-based enzyme-linked immunosorbent assay (ELISA-G), the AMDV capsid protein-based ELISA (ELISA-P), counterimmunoelectrophoresis (CIEP), and the iodine agglutination test (IAT). Among them, CIEP has been considered a gold standard [9]. Since controlling AD has not succeeded because no effective vaccine or medicine has been created, culling mink that have tested positive using CIEP has been applied as the primary method to control AD [10,11,12,13]. However, implementing the culling method following a positive diagnosis using CIEP requires screening a large number of animals, consequently causing the demand for an additional labor force and problems in the availability of the CIEP antigen [14]. Therefore, predicting AD without using CIEP will provide an effective way for controlling AD in mink farms.

Machine learning (ML) algorithms have proven successful in diagnosing and predicting diseases [15,16] and are becoming more popular in health care. They are also widely used methods in agriculture [17,18,19], such as for high throughput phenotyping [20] or predicting plant diseases [21]. In animals, the ML algorithms have been used for monitoring the health status [22,23], product quality [24,25], and prediction of diseases [26,27,28,29,30]. Selection of the ML algorithms for studies on farm animals depends on the traits and data, and their performance also varies among the studies [17,28,31]. For instance, different ML algorithms, including artificial neural networks [29], support vector machines [31], or random forest [28], had an accuracy of 85–90% for the classification of mastitis in dairy cows. Overall, ML is appropriate for large data or data with many predictors, missing values, and abnormal distributions. Data from a mink farm often has many predictors and missing values that might benefit from the application of ML. For instance, ML can be used for predicting some phenotypes such as daily feed consumption and reproductive performance or for recognizing the missing labels in the animal IDs in the mink farms. Therefore, we examined the performance of nine ML algorithms including artificial neural networks, decision tree, extreme gradient boosting, gradient boosting method, K-nearest neighbors, linear discriminant analysis, linear support vector machines, naive bayes, and random forest for the classification of AD in American mink. We used 33 different features obtained from different data sources in an AD positive mink farm for the classification of AD-infected mink. We used four different matrices for assessing the performance of the ML algorithms. Finally, we performed the Wilcoxon test to examine if these ML algorithms are significantly different.

## 2. Materials and Methods

The animals used in this study were raised according to the Code of Practice for the Care and Handling of Farmed Mink guidelines published by the Canada Mink Breeders Association (https:/www.nfacc.ca/pdfs/codes/mink_code_of_practice.pdf, accessed on 20 October 2019). The animal care was followed by protocols approved by the Dalhousie University Animal Care and Use Committee (certification# 2018-009 and 2019-012).

### 2.1. Animals and the Phenotypic Records

**Figure 1:**
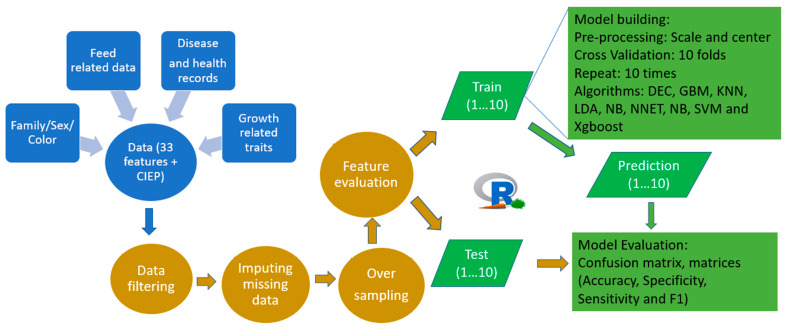
A simplified workflow representation of the current study.

The mink used in this study were raised under standard farming conditions at the Canadian Centre for Fur Animal Research (CCFAR) at Dalhousie University, Faculty of Agriculture (Truro, NS, Canada) from 2013 to 2019. All mink had ad libitum access to feed and water. The feeds were adjusted based on the animals’ needs in each production period, with a higher dry matter added during the growing and furring periods [32]. Three AD tests were conducted using the established protocols described by Hu et al. [33]. In brief, the blood samples of the mink (1157 individuals) were collected using the toenail clipping approach. Both the ELISA-G and ELISA-P systems were employed to quantify the anti-AMDV antibodies in the serum. The ELISA-G scores (0–7) and ELISA-P scores (0–8) were conducted at Middleton Veterinary Services (Middleton, NS, Canada), and Nederlandse Federatie van Edelpelsdierenhouders (Wijchen, The Netherlands), respectively. The CIEP tests were conducted at the Animal Health Laboratory at the University of Guelph (Guelph, ON, Canada) to detect the existence of anti-AMDV antibodies in the blood samples, and the results were recorded as negative or positive. The IATs were conducted at the CCFAR to measure the serum gamma globulin level in the serum, and the results were scored into four categories from 0 (low) to 4 (high). All bodyweight (BW), growth parameters, and feed intake data were collected using the established protocols described by Do et al. [34] and Davoudi et al. [35], respectively. The mink were housed individually in single cages, and the feed was distributed to each pen every morning. The amount of allocated feed was regulated based on the leftover records one day before in order to avoid unnecessary feed waste and to meet the mink’s appetite. The daily feed intake (DFI) was obtained by subtracting the amount of leftover from the quantity of feed supplied. The average daily feed intake (ADFI) was calculated by averaging the DFI records obtained during the test period. The average daily gain (ADG), feed conversion ratio (FCR), Kleiber ratio (KR), residual feed intake (RFI), residual gain (RG), and residual intake and gain (RIG) were derived from the body weight and daily feed intake data [35]. The growth curve parameters, including asymptotic weight (α), growth rate at mature (k), shape parameter (m), weight at the inflection point (WIP), and age at the inflection point (AIP), were obtained from the body weights of mink using the Richard growth model [34]. A total of 33 features were examined for the development of the ML algorithms to classify animals for AD. The number of CIEP positive and negative mink as well as the mean values for these features are given in Table 1. A simplifized workflow of the current study is shown in Figure 1.

### 2.2. Algorithm Selection and the Data Preparation

The classification of AD-infected mink was constructed using the following algorithms: artificial neural networks, decision tree, extreme gradient boosting, gradient boosting, K-nearest neighbors, linear discriminant analysis, support vector machines (linear form), naive bayes, and random forest. These algorithms were selected as they have been widely used for the diagnosis of human diseases, e.g., cancers [36,37,38], as well as for predicting phenotypes in livestock [17,19,39]. All calculations were performed in R using the caret package [40], and CIEP was used as the response variable in the models. Since the CCFAR was infected with AD in 2012, more animals were positive (954) than negative (203) using CIEP in the current dataset. The missing data of features (Table 1) were input using the mice R package [41]. If the imbalance ratio is high, the decision function favors the majority group (positive CIEP group). For non-probabilistic classifiers such as logistic regression, neural networks, and support vector machines algorithms, an imbalanced data structure can affect their parameters [39]. We used the over-sampling function in the Rose package [42] to create the balanced data. In this function, the minority group (negative CIEP, *n* = 203) was oversampled from 203 to 954 in order to balance a sample size as the majority group (positive CIEP, *n* = 954). The preProcess function from the caret package [40] was used to scale and center the variables in the training dataset. The relative importance of the features and feature selection were examined using the Boruta package [43]. The Boruta algorithm is a wrapper approach that is built based on the random forest. This algorithm creates shadow features as a replica of actual features, and then randomly shuffles to remove any correlation with the response variable. In the next step, a random forest classifier is run, and the Z-score is computed by dividing the average loss by its standard deviation. The maximum Z-score of randomized shadow features is used to set a threshold for the selection of important features [43]. If the Z-score computed for an actual feature is significantly more than the Z-score of the shadow feature, then it is considered as an important feature [43].

### 2.3. Model Training and Performance Assessment

Following the oversampling, the data was randomly divided into 80% for training and 20% for testing datasets. We created ten different sets of training-testing dataset using the createDataPartition function. The models were built in each training dataset and evaluated in each test data. In the training dataset, we used the trainControl function to select the hyperparameters for the model building. The repeated cross-validation methods implemented in the trainControl function were used. In this method, for each one of the ten iterations, the hyperparameters were selected using a search within the 10-fold cross-validation structure on a random 70% subset of the training dataset. Each algorithm was run separately using the default initial hypermeters and the train function of the caret package. The confusionMatrix function was used to evaluate the model performance from the best built model for each training dataset and the corresponding testing dataset.

The model fit and ranking of the models were assessed using several scores that were computed using the number of true positive (TP), true negative (TN), false positive (FP), and false-negative (FN). The following formulas were used for the calculation of accuracy (Equation (1)), specificity (Precision; Equation (2)), which is the fraction of correct predictions, sensitivity (Recall; Equation (3)), which measures a fraction of the correct predictions per true number of samples, and F-Measure (F1; Equation (4)), which is a goodness of fit assessment for a classification analysis that balances precision and recall:

(1) $$\mathrm{Accuracy}=\frac{\left(\mathrm{TP}\;+\;\mathrm{TN}\right)}{\left(\mathrm{TP}\;+\;\mathrm{TN}\;+\;\mathrm{FP}\;+\;\mathrm{FN}\right)}$$

(2) $$\mathrm{Specificity}\;\left(\mathrm{Precision}\right)=\frac{\mathrm{TP}}{\left(\mathrm{TP}\;+\;\mathrm{FP}\right)}$$

(3) $$\mathrm{Sensitivity}\;\left(\mathrm{Recall}\right)=\frac{\mathrm{TP}}{\left(\mathrm{TP}\;+\;\mathrm{FN}\right)}$$

(4) $$F1\;=\;\frac{2\times \left(\mathrm{Precision}\;\times \;\mathrm{Recall}\right)}{\left(\mathrm{Precision}\;+\;\mathrm{Recall}\right)}$$

A receiver operating characteristic (ROC) curve was used to depict the sensitivity against 1-specificity over all possible decision thresholds ranging for classifying the predicted AD-infected mink and was characterized using the *pROC* package [44]. The accuracy of the models was assessed by calculating the area under the curve (AUC). The values of AUC were interpreted as non-accurate (AUC = 0.5), less accurate (0.5 < AUC ≤ 0.7), moderately accurate (0.7 < AUC ≤ 0.9), highly accurate (0.9 < AUC < 1) and perfectly (AUC = 1) [45]. Moreover, the pairwise differences in the accuracy of the models were compared using the Wilcoxon test.

## 3. Results and Discussion

### 3.1. Feature Importance and the Model Performance

**Figure 2:**
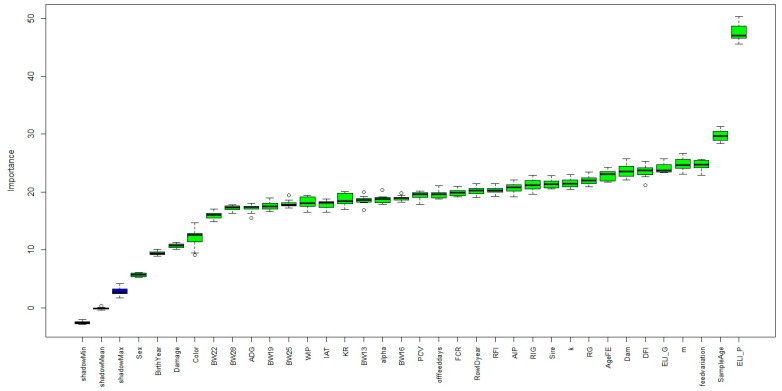
Feature importance based on the Boruta package. Blue boxplots correspond to the minimal, average, and maximum Z scores of a shadow attribute. Green boxplots represent the Z scores of the confirmed attributes. ° in the plot indicated the outliners.

The descriptive statistics of all numerical features are shown in Table 1. A total of 33 different features were collected and used as input in the Boruta package. The relative importance of the features based on the random forest from the Boruta package is shown in Figure 2.

The ELISA-P was identified as the most important feature for the classification of AD-infected mink. Other important features based on the ranking by the Boruta package for the AD-infected mink classification were those related to bodyweight measures, growth curve parameters, and DFI. Sex, birth year, and color were the less important features for the classification of AD-infected mink. The importance of the ELISA is expected as the ELISA systems are also alternative methods for the diagnosis of AD [46]. Previously, we also reported that the ELISA tests had significant phenotypic and genetic correlations with CIEP [33]. The age at sampling might be an important feature for CIEP since, if animals stayed on the farm for a longer period of time, they might have a higher chance of being infected by the AMDV. Bodyweights, growth curve parameters, and DFI were important traits for the growth of animals. Since AD harms the animal’s health and growth [47], it was expected that these features would be important for classifying AD-infected animals. Interestingly, the variation in feed intake was important for the CIEP classification, which might be because of the inconsistency in the diet of infected mink. Sex and color type were less important for the CIEP classification, which was also supported by our previous study that these effects were not significantly affecting the CIEP [33].

### 3.2. Performance Assessment

Table 2 presents the sensitivity, specificity, F1, and accuracy of the nine ML algorithms using four different sub-sampling procedures. Overall, the sensitivity, specificity, F1, and accuracy were varied between the algorithms. The specificity ranged from 0.588 (K-nearest neighbors) to 0.938 (random forest), while the sensitivity ranged from 0.841 (naive bayes) to 0.987 (extreme gradient boosting). All algorithms obtained higher specificity values than sensitivity values. All algorithms had the F1 and accuracy values of more than 0.7, thereby indicating that they could be used for the classification of AD-infected mink with an acceptable accuracy. The random forest algorithm had an excellent performance considering both the F1 measure and accuracy (>0.95).

Table 3 shows the confusion matrix obtained from the random forest algorithm. The random forest could correctly classify 186 out of 190 CIEP positive mink and 184 out of 190 CIEP negative mink. The Friedman test indicated the significant differences in the accuracies obtained from the different algorithms according to the subsampling procedures (*p*-value < 2.2 × 10^−16^). The paired samples used in the Wilcoxon tests for the differences indicated that all algorithms had significant differences in their accuracies, except for the K-nearest neighbors with the linear discriminant analysis (*p* = 0.18) and the naive bayes (*p* = 0.51) (Table 4).

**Figure 3:**
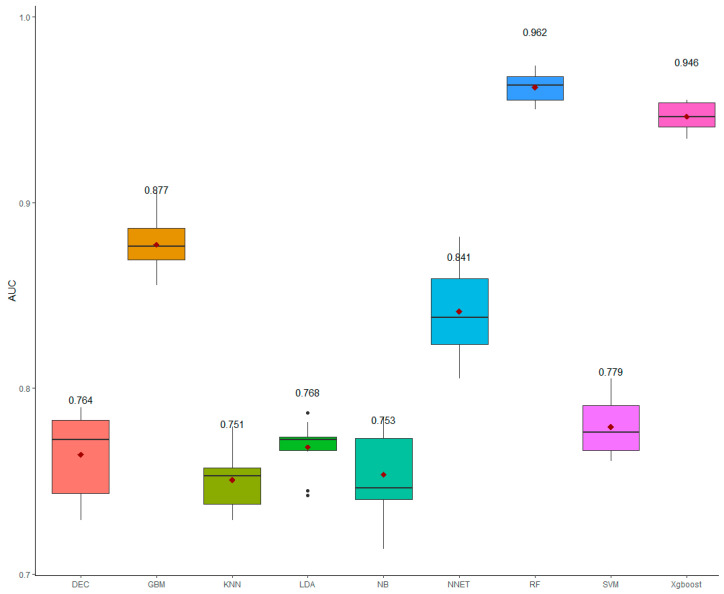
The boxplot for the area under the ROC curve (AUC) for the nine machine learning algorithms*. The values indicated the mean of AUC obtained from tenfold cross-validation. * Decision tree (DEC), gradient boosting (GBM), K-nearest neighbors (KNN), linear discriminant analysis (LDA), naive bayes (NB), artificial neural networks (NNET), random forest (RF), linear support vector machines (SVM), and extreme gradient boosting (Xgboost). The red diamond shapes indicated the mean of AUC; the black dots indicated the outliners.

The average values of the AUC (Figure 3) indicated that the random forest and the extreme gradient boosting were the best algorithms with the highest AUC as their AUC values were >0.90. Other algorithms had a moderate AUC with the AUC values ranging from 0.75 (naive bayes and K-nearest neighbors) to 0.88 (gradient boosting).

All of the tested algorithms in this study have been used for the diagnosis of diseases [17,39,45]. The decision tree, gradient boosting, random forest, and extreme gradient boosting are all tree-based models, and their performances will be influenced by the class imbalances via the leaf impurity. To solve the problem of class imbalance, the oversampling method was chosen for handling the imbalance classifiers as it does not lead to any information loss. In the current study, the random forest outperformed the other methods, which was consistent with the previous study [39] that implemented the random forest to predict the leg weakness in pigs. However, the random forest was not the best method as observed by Shao et al. [48], who showed that the support vector machines outperformed the neural networks, random forest, and linear regression to predict the corrected inventory decision for the market using China’s hog inventory data. The random forest was also less accurate compared with the support vector machines, kernel ridge regression, and Adaboost.R2 in the prediction of the reproductive performance traits in pigs using genomic data [49]. The random forest approach is known to be fairly stable in the presence of outliers and noise and can handle the correlations between the predictors [49,50].

Extreme gradient boosting was the second-best method for the classification of AD-infected mink, which might be because this method could perform implicit variable selections and could capture the non-linear relationships [51,52]. Both the random forest and the extreme gradient boosting showed great potential for the classification of CIEP in the current study with high accuracies, F1 values, and AUCs. Especially, these algorithms were close to perfect (sensitivity > 0.99) in classifying AD-infected mink. Both the random forest and extreme gradient boosting succeeded in detecting the posture and behavior in dairy cows [51] with the accuracy obtained from the extreme gradient boosting algorithm in predicting the posture was 0.99, and the random forest had the highest overall accuracy in predicting the behavior (0.76). The NNET performed fairly well, but was not the best, and that could be due to the limitation of the fine tuning in hyper parameters for the NNET or the limitation of the small sample size in the current study. Nevertheless, the performance of the ML algorithms depends on the data, and therefore, it is necessary to test different algorithms to find the most suitable one.

The current study had some limitations. Although CIEP has been adapted by the mink farmers for controlling AD, it is important to mention that the CIEP test results are binary outcomes while AD is a chronic disease. The CIEP measure is sensitive to the status of the disease; therefore, the results of the current study might be limited by the lack of repeated measurements of CIEP. More frequent measures of CIEP are required to confirm the AD status better and to consequently apply the ML algorithms to the classification of AD-infected mink. Additionally, even though the random forest reached a very high accuracy, specificity, and sensitivity, some individuals were still wrongly classified. Therefore, larger sample sizes with more features or better hyper-parameterizing are required for correctly classifying these individuals. The results of the current study were also limited to use in the AD positive mink farms. However, the majority of mink farms are infected with AD; thus, the results are still beneficial for most mink farmers. In the meantime, these results could be helpful for the farmers who want to cull animals based on the classified AD-infected mink obtained by the ML algorithms.

Finally, although being considered as a gold standard for the AD test, CIEP is a relatively expensive test and requires a large labor force, due to the many steps in the CIEP test that are performed manually. Moreover, the CIEP results are prone to false-positive results as the accuracies of the CIEP results are dependent on the experience of the readers in visualizing the bands. These drawbacks of CIEP limit its application in large mink farms. Alternatively, the ELISA test can be used for high-throughput assays, and the ELISA results can be used in the ML approaches (e.g., random forest or extreme gradient boosting) to accurately classify the AD-infected mink. Therefore, the mink farmers might not need to perform the CIEP test, but use the information from the ELISA test to predict AD risks and to decide which animals are needed to be culled for the control of AD.

## 4. Conclusions

In summary, among the nine ML algorithms, the random forest was the best method for the classification of AD-infected mink in the current dataset. This study indicated that it is possible to classify AD-infected mink with a high accuracy, specificity, and sensitivity using the random forest algorithms. Therefore, it is suggested that the random forest algorithm might be used for classifying the AD-infected mink in other AD-positive farms. Given the fact that the current study used the data from only one AD-positive farm and the performance of the ML algorithms were sensitive to the data input, it is recommended that further model tests in other AD-positive farms be performed. Since AD is a chronic disease, it is also recommended to collect disease records more frequently for better disease monitoring. Finally, it is also recommended to combine the genomic information to optimize the model for the implementation of machine learning methods in controlling AD.

## Figures and Tables

**Table 1:** A summary of features used for the classification of CIEP.

		CIEP Negative			CIEP Positive		
Features	Name	N	Mean	SD	N	Mean	SD
General features							
Sample_Age (day)	Age at the collection of blood for AD testing	201	197.3	63.1	902	234.6	136.3
RowIDyear	Row number where the mink were kept each year	200	6.55	2.80	853	5.20	3.09
AgeFE	Age of the mink (in days) when the feeding measure started	200	198.25	3.50	852	197.60	9.73
Damage	The damage score in the fur	202	1.42	0.63	881	1.34	0.57
Aleutian disease and health-related tests							
Elisa_P	In vitro cultured Aleutian mink disease virus antigen-based enzyme-linked immunosorbent assay test	203	0.33	0.97	924	1.67	2.29
Elisa_G	Capsid protein of Aleutian mink disease virus-based enzyme-linked immunosorbent assay test	203	0.64	0.98	922	2.46	2.19
PCV	Packed cell volume	201	58.11	2.94	919	56.67	4.03
IAT	Iodine agglutination test	200	0.42	0.60	918	0.77	1.05
Feed intake and efficiency							
DFI	Daily feed intake	200	221.36	56.24	853	227.21	57.45
ADG	Average Daily Gain	199	7.45	3.40	821	8.60	3.86
FCR	Food Conversion Ratio	199	33.59	11.17	813	30.42	11.40
RFI	Residual feed intake	198	7.45	34.69	820	−1.14	36.96
RG	Residual intake and gain	199	0.11	1.12	817	−0.01	1.44
RIG	Residual gain	198	−0.14	1.37	819	0.01	1.56
KR	Kleiber ratio	199	5.19	1.40	821	5.51	1.58
Offfeeddays	Proportion of off-feed days based on feed intake	199	0.05	0.09	842	0.06	0.08
Feedvariation	Day-to-day variation in feed intake	199	48.22	11.55	842	49.15	18.02
Body weight and growth parameters							
BW13	Body weight at week 13	198	1.15	0.26	837	1.27	0.30
BW16	Body weight at week 16	198	1.42	0.36	834	1.58	0.41
BW19	Body weight at week 19	198	1.62	0.46	829	1.82	0.53
BW22	Body weight at week 22	198	1.82	0.54	824	2.04	0.61
BW25	Body weight at week 25	198	1.88	0.56	810	2.13	0.64
BW28	Body weight at week 28	197	1.92	0.59	805	2.17	0.67
Alpha	Weight at maturity [34]	198	1.99	0.63	807	2.25	0.71
k	Maturation rate [34]	198	0.24	0.10	803	0.24	0.11
m	Inflection parameter [34]	198	0.68	0.90	798	0.64	0.83
AIP	Age at the inflection point [34]	195	10.79	1.86	799	10.97	1.83
WIP	Weight at the inflection point [34]	196	0.89	0.33	804	1.01	0.35

SD: Standard Deviation.

**Table 2:** The mean (SE *) of the sensitivity, specificity, F1, and accuracy of the classification of Aleutian disease using nine machine learning algorithms.

Algorithms	Sensitivity	Specificity	F1	Accuracy
Artificial Neural Networks	0.805 ± 0.008	0.877 ± 0.016	0.836 ± 0.096	0.841 ± 0.007
Decision tree	0.634 ± 0.007	0.894 ± 0.014	0.726 ± 0.01	0.764 ± 0.007
Extreme Gradient Boosting	0.905 ± 0.002	0.987 ± 0.005	0.944 ± 0.002	0.946 ± 0.002
Gradient Boosting	0.831 ± 0.005	0.924 ± 0.01	0.871 ± 0.035	0.877 ± 0.004
K-Nearest Neighbors	0.588 ± 0.006	0.909 ± 0.011	0.700 ± 0.000	0.749 ± 0.005
Linear Discriminant Analysis	0.672 ± 0.005	0.865 ± 0.009	0.743 ± 0.001	0.768 ± 0.004
Naive Bayes	0.666 ± 0.007	0.841 ± 0.014	0.73 ± 0.002	0.753 ± 0.007
Random Forest	0.938 ± 0.003	0.986 ± 0.005	0.961 ± 0.088	0.962 ± 0.002
Support Vector Machines	0.687 ± 0.005	0.872 ± 0.01	0.757 ± 0.001	0.779 ± 0.004

* SE: Standard errors.

**Table 3:** A confusion matrix obtained from the random forest algorithm for the classification of CIEP in mink.

		**Actual Data**		$\mathrm{Accuracy}=\frac{186\;+\;184}{186\;+\;4\;+\;6\;+\;184}=0.974$ $F1=\frac{2\times 0.979\;\times \;0.968}{0.979\;+\;0.968}=0.973$
		**Positive**	**Negative**	
Predicted data	Positive	184	4	
	Negative	6	186	
	Total	190	190	
		$\mathrm{Sensitivity}=\frac{184}{190}=0.968$	$\mathrm{Specificity}=\frac{186}{190}=0.979$	

**Table 4:** The paired samples used in the Wilcoxon tests for the differences in the accuracies obtained from the nine machine learning algorithms.

ModelA *	ModelB	FDR **	ModelA	ModelB	FDR
GBM	DEC	7.90 × 10^−18^	RF	KNN	7.90 × 10^−18^
KNN	DEC	2.36 × 10^−3^	SVM	KNN	1.18 × 10^−7^
LDA	DEC	5.15 × 10^−3^	XGBOOST	KNN	7.90 × 10^−18^
NB	DEC	8.90 × 10^−6^	NB	LDA	9.33 × 10^−3^
NNET	DEC	1.91 × 10^−17^	NNET	LDA	1.10 × 10^−17^
RF	DEC	7.90 × 10^−18^	RF	LDA	7.90 × 10^−18^
SVM	DEC	1.18 × 10^−4^	SVM	LDA	1.51 × 10^−12^
XGBOOST	DEC	7.90 × 10^−18^	XGBOOST	LDA	7.90 × 10^−18^
KNN	GBM	7.90 × 10^−18^	NNET	NB	1.10 × 10^−17^
LDA	GBM	7.90 × 10^−18^	RF	NB	7.90 × 10^−18^
NB	GBM	7.90 × 10^−18^	SVM	NB	8.91 × 10^−12^
NNET	GBM	9.37 × 10^−13^	XGBOOST	NB	7.90 × 10^−18^
RF	GBM	7.90 × 10^−18^	RF	NNET	7.90 × 10^−18^
SVM	GBM	7.90 × 10^−18^	SVM	NNET	2.23 × 10^−17^
XGBOOST	GBM	7.90 × 10^−18^	XGBOOST	NNET	7.90 × 10^−18^
LDA	KNN	0.18	SVM	RF	7.90 × 10^−18^
NB	KNN	0.51	XGBOOST	RF	3.77 × 10^−8^
NNET	KNN	1.08 × 10^−17^	XGBOOST	SVM	7.90 × 10^−18^

* Decision tree (DEC), gradient boosting (GBM), K-nearest neighbors (KNN), linear discriminant analysis (LDA), naive bayes (NB), artificial neural networks (NNET), random forest (RF), support vector machines (SVM), and extreme gradient boosting (Xgboost). ** FDR: The *p*-value adjusted for false discovery rate.

## Data Availability

The datasets used in this work are available from the corresponding author upon academic request.
